# Supplementary material for: Identification of photoperiod-induced specific miRNAs in the adrenal glands of Sunite sheep (Ovis aries)
Source: Front Vet Sci. 2022 Jul 22;9:888207. doi: 10.3389/fvets.2022.888207 (PMC9354845; doi:10.3389/fvets.2022.888207)
Supplement: Supplementary file 1 [file Data_Sheet_1.ZIP › Supplementary file/miRDeep2 file.zip!/miRDeep2 file/Novel_Structure/SP42A3/Novel_189.pdf]

Provisional ID	:	NC_019475.2_24691
Score total	:	64.9
Score for star read(s)	:	3.9
Score for read counts	:	57.2
Score for mfe	:	2.2
Score for randfold	:	1.6
Score for cons. seed	:	
Total read count	:	124
Mature read count	:	112
Loop read count	:	0
Star read count	:	12

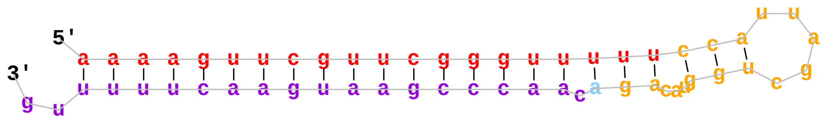

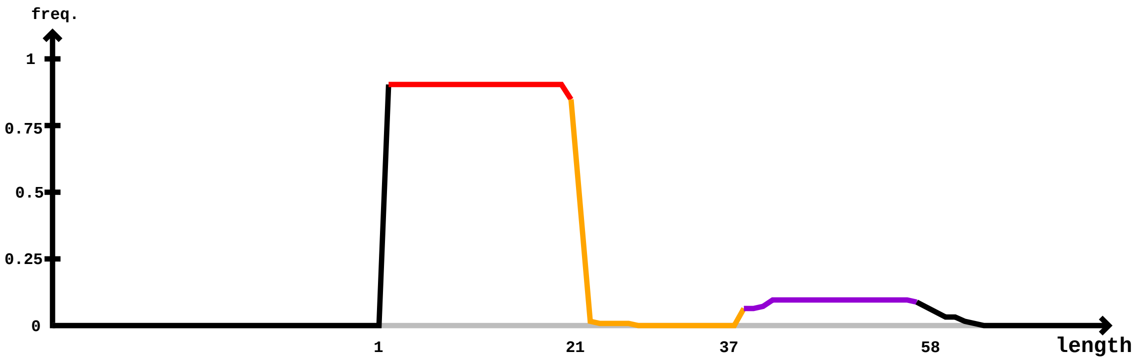

Mature Star

```
5'- ccuggggaggaagggauguacuggguuggcccaaaaguucguucggguuuuuccauuagcugguacagacaacccgaaugaacuuuuuggccaauucaauauuucca   -3'   obs
    ccuggggaggaagggauguacuggguuggcccaaaaguucguucggguuuuuccauuagcugguacagacaacccgaaugaacuuuuuggccaauucaauauuucca         exp
    (((.....)))...(((.(((.((((((((((.(((((((((((((((((((((((.....)))...))).))))))))))))))))).)))))))))).))).))).   reads   mm   sample
    ................................aaaaguucguucggguuuu........................................................   7       0    seq
    ................................aaaaguucguucggguuuuu.......................................................   104     0    seq
    ................................aaaaguucguucggguuuuucc.....................................................   1       0    seq
    ................................aaaaguucguucggguuuuuccauua.................................................   1       0    seq
    .....................................................................caacccgaaugaacuuuu....................   1       0    seq
    .....................................................................caacccgaaugaacuuuuu...................   1       0    seq
    .....................................................................caacccgaaugaacuuuuug..................   4       0    seq
    .....................................................................caacccgaaugaacuuuuugg.................   2       0    seq
    .......................................................................acccgaaugaacuuuuuggcA...............   1       1    seq
    ........................................................................cccgaaugaacuuuuuggcA...............   1       1    seq
    ........................................................................cccgaaugaacuuuuuggccG..............   1       1    seq
    ........................................................................cUcgaaugaacuuuuuggccaa.............   1       1    seq
```
